# Background frequency can enhance the prognostication power of EEG patterns categories in comatose cardiac arrest survivors: a prospective, multicenter, observational cohort study

**DOI:** 10.1186/s13054-021-03823-y

**Published:** 2021-11-17

**Authors:** Youn-Jung Kim, Min-Jee Kim, Yong Hwan Kim, Chun Song Youn, In Soo Cho, Su Jin Kim, Jung Hee Wee, Yoo Seok Park, Joo Suk Oh, Dong Hoon Lee, Won Young Kim, the Korean Hypothermia Network Investigators, Ji Hoon Kim, Kyu Nam Park, Won Jung Jeong, Seung Pill Choi, Mi Jin Lee, Jong-Seok Lee, Su Jin Kim, Tae Chang Jang, Inbyung Kim, Yong Hwan Kim, Won Young Kim, Jonghwan Shin, Ji Hwan Lee, Hyung Jun Moon, Giwoon Kim, Wook-jin Choi, Joo Suk Oh, Chul Han, Byung Kook Lee, Taeoh Jeong, Dong Hoon Lee, Min Jin Hong, Gyu Chong Cho, Young Hwan Lee, Youdong Sohn, In Soo Cho, Je Sung You, Changsun Kim, Kyoung-Chul Cha, Soo Hyung Cho

**Affiliations:** 1 Department of Emergency Medicine, Asan Medical Center, Ulsan University College of Medicine, Seoul, Korea; 2 Department of Pediatrics, Asan Medical Center Children’s Hospital, Ulsan University College of Medicine, Seoul, Korea; 3 Departments of Emergency Medicine, Samsung Changwon Hospital, Sungkyunkwan University School of Medicine, Changwon, Korea; 4 Department of Emergency Medicine, Seoul St. Mary’s Hospital, College of Medicine, The Catholic University of Korea, Seoul, Korea; 5 Department of Emergency Medicine, Hanil General Hospital, Seoul, Korea; 6 Department of Emergency Medicine, Korea University College of Medicine, Seoul, Korea; 7 Department of Emergency Medicine, Yeouido St. Mary’s Hospital, The Catholic University of Korea College of Medicine, Seoul, Korea; 8 Department of Emergency Medicine, Yonsei University College of Medicine, Seoul, Korea; 9 Department of Emergency Medicine, Uijeongbu St. Mary’s Hospital, The Catholic University of Korea College of Medicine, Uijeongbu-si, Korea; 10 Department of Emergency Medicine, Chung-Ang University, College of Medicine, Seoul, Korea

**Keywords:** Out-of-hospital cardiac arrest, Electroencephalography, Prognosis, Neurologic outcome, Targeted temperature management

## Abstract

**Background:**

We assessed the prognostic accuracy of the standardized electroencephalography (EEG) patterns (“highly malignant,” “malignant,” and “benign”) according to the EEG timing (early vs. late) and investigated the EEG features to enhance the predictive power for poor neurologic outcome at 1 month after cardiac arrest.

**Methods:**

This prospective, multicenter, observational, cohort study using data from Korean Hypothermia Network prospective registry included adult patients with out-of-hospital cardiac arrest (OHCA) treated with targeted temperature management (TTM) and underwent standard EEG within 7 days after cardiac arrest from 14 university-affiliated teaching hospitals in South Korea between October 2015 and December 2018. Early EEG was defined as EEG performed within 72 h after cardiac arrest. The primary outcome was poor neurological outcome (Cerebral Performance Category score 3–5) at 1 month.

**Results:**

Among 489 comatose OHCA survivors with a median EEG time of 46.6 h, the “highly malignant” pattern (40.7%) was most prevalent, followed by the “benign” (33.9%) and “malignant” (25.4%) patterns. All patients with the highly malignant EEG pattern had poor neurologic outcomes, with 100% specificity in both groups but 59.3% and 56.1% sensitivity in the early and late EEG groups, respectively. However, for patients with “malignant” patterns, 84.8% sensitivity, 77.0% specificity, and 89.5% positive predictive value for poor neurologic outcome were observed. Only 3.5% (9/256) of patients with background EEG frequency of predominant delta waves or undetermined had good neurologic survival. The combination of “highly malignant” or “malignant” EEG pattern with background frequency of delta waves or undetermined increased specificity and positive predictive value, respectively, to up to 98.0% and 98.7%.

**Conclusions:**

The “highly malignant” patterns predicted poor neurologic outcome with a high specificity regardless of EEG measurement time. The assessment of predominant background frequency in addition to EEG patterns can increase the prognostic value of OHCA survivors.

*Trial registration* KORHN-PRO, NCT02827422. Registered 11 September 2016—Retrospectively registered.

**Supplementary Information:**

The online version contains supplementary material available at 10.1186/s13054-021-03823-y.

## Background

Accurate neurological prognostication is critically important in cardiac arrest survivors with ischemic/reperfusion brain injury. Late awakening of comatose out-of-hospital cardiac arrest (OHCA) patients should be distinguished from irreversible brain damage. A multimodal approach involving delayed timing (after > 72 h) of prognostication has been recommended to minimize the possibility of inappropriate withdrawal of life-sustaining therapy (WLST) for patients who may otherwise achieve meaningful neurological recovery [1, 2].

Electroencephalography (EEG) is the most widely used tool in clinical practice to evaluate cortical brain activity and diagnose seizure [3]. Its use as a neuroprognostic tool is promising, but the inter-rater variability and definitions used to describe specific findings and patterns particularly in post-cardiac arrest patients are limited [4–8]. The American Clinical Neurophysiology Society (ACNS) proposed a standardized terminology for critical care EEG [9]. Current ERC guidelines suggest using highly malignant EEG defined as suppressed background with or without periodic discharges and burst-suppression for indicators of a poor prognosis [2]. Westhall et al. proposed three standardized EEG pattern categories (“highly malignant,” “malignant,” and “benign”) for the assessment of neurological outcome [10]. Recently, a sub-study of the Targeted Temperature Management (TTM) trial first validated the “highly malignant” EEG pattern and reported that it accurately predicts poor neurological outcome with high specificity (98–100%) in EEGs recorded at a median time of 77 h (range 53–102) after cardiac arrest [11]. This was confirmed with the findings of a very recent study of 62 patients that the presence of a “highly malignant” EEG pattern was predictive of a poor neurological outcome, with 100% specificity and 42% sensitivity [12]. However, the new American Heart Association guidelines did not include these three EEG patterns, indicating that their prognostic performance needs to be validated in a large cohort. Moreover, the prognostic accuracy of the “malignant” pattern has been questionable, and the prognostic value of these standardized EEG pattern categories based on the timing of EEG has not been determined yet.

This study aimed to assess the prognostic performance of the three standardized EEG pattern categories according to the EEG timing (early vs. late) using a multicenter, prospective registry of out-of-hospital cardiac arrest (OHCA) patients treated with TTM. We also determined the EEG features that can improve the predictive power for poor neurologic outcomes at 1 month after cardiac arrest.

## Methods

### Study design and patients

This multicenter, prospective, observational registry-based study was performed using the Korean Hypothermia Network prospective registry (KORHN-PRO) data between October 2015 and December 2018. Among the 20 hospitals that participated in the KORHN-PRO, we extracted data from 14 tertiary care university-affiliated teaching hospitals, which could provide high-quality standardized intermittent EEG data during the post-resuscitation period. The institutional review board of all participating hospitals reviewed and approved the study protocol, including the institutional review board of Asan Medical Center (No. 2019-1204) and the investigators obtained written informed consent from all patients' legal surrogates. The KORHN-PRO registry was registered under clinicaltrials.gov as protocol NCT02827422.

We included all adult comatose patients (aged $\ge$18 years) with successfully resuscitated non-traumatic OHCA who were treated with TTM between October 2015 and December 2018 and who underwent standard intermittent EEG within 7 days after return of spontaneous circulation (ROSC). The registry excluded OHCA patients with terminal illness, i.e., a life expectancy of < 6 months, as documented in the medical records, under hospice care, with a pre-documented “Do Not Resuscitate” card, with intracranial bleeding or acute stroke, and with pre-arrest cerebral performance category (CPC) score 3 or 4. We excluded patients without EEG data within 7 days after ROSC or with poor-quality EEG data. Throughout the study period, an initial standard EEG examination was recommended to be performed as soon as possible for OHCA survivors for the detection of seizure activity in the early stages of TTM. However, the EEG timing was based on the practical availability of each hospital.

This study categorized early and late EEG examination according to the timeline for neurological recovery provided the 2011 consensus statement from the American Heart Association on outcome measures for resuscitation research: the post-resuscitation phase (< 72 h after ROSC), the early hospitalization phase (72 h–7 days after ROSC) [13]. Early EEG was defined as EEG performed within 72 h after ROSC, whereas late EEG was defined as EEG performed between 72 h and 7 days. For those who underwent EEG examination two or more times, we used the first EEG examination for analysis. All patients were observed for 1 month after cardiac arrest by neurologic assessments according to their CPC score, and the independent data input commission followed up the patients to 6 months after cardiac arrest.

### Patient management

All patients received post-resuscitation care according to the then-current advanced cardiac life support guidelines [14]. Cooling devices, such as Blanketrol II (Cincinnati Subzero Products, Cincinnati, OH, USA), Arctic Sun Energy Transfer Pad (Medivance Corp., Louisville, CO, USA), or an endovascular cooling device (Thermoguard; ZOLL Medical Corporation, Chelmsford, MA, USA), were used to maintain the target temperature (32–36 °C) for 24 h for the patients. The patients were rewarmed at a rate of 0.25–0.5 °C/hour after 24 h and were monitored to maintain normothermia (37 °C) for 72 h after ROSC. Sedatives and analgesics including propofol, remifentanil, morphine, midazolam, and fentanyl were used, and a neuromuscular blocking agent was administered to control shivering or respiratory dyssynchrony between the ventilator and the patient. Valproate or levetiracetam was administered when the seizure activity was detected on EEG examination. All patients received standard intensive care according to institutional protocols. WLST was legally prohibited in South Korea until February 2018, and all patients in this study received treatment at the institution until death or recovery.

### EEG examination and pattern classification

Standard EEG examination was performed with a 21-electrode setup according to the international 10–20 electrode system (Fp1-2, F7-8, T7-8, P7-8, F3-4, C3-4, P3-4, O1-2, Fz, Cz, Pz), with a sampling rate of 200 Hz and a high-pass filter of 0.1-Hz for 15–30 min. For this study, a board-certified epileptologist (M.K.), who was blinded to all clinical information, reviewed the original EEG recording. The EEG patterns were categorized into one of the three groups: “highly malignant,” “malignant,” and “benign” patterns (see Additional file 1) [8, 10]. Highly malignant patterns include suppressed background (amplitude < 10 μV) without discharges or with continuous periodic discharges and burst-suppression background with or without discharges [8, 10]. Malignant pattern was defined as EEG with any malignant feature including malignant periodic or rhythmic patterns (abundant periodic discharges; abundant rhythmic polyspike-/spike-/sharp-and-wave; unequivocal electrographic seizure); malignant background (discontinuous background; low-voltage background, defined as amplitude 1020 μV; reversed anterior–posterior gradient); and unreactive EEG (absence of background reactivity or only stimulus-induced discharges) [8, 10]. However, we excluded unreactive EEG from the “malignant” pattern owing to no generally acknowledged standard for reactivity testing [2, 11, 15]. Benign EEG pattern was defined as continuous normal-voltage EEG without any malignant features [8, 10]. In addition to EEG pattern classification, the predominant frequency of background EEG was assessed and categorized into alpha, theta, delta waves, and undetermined background frequency (see Additional file 2) [9, 16]. An undetermined frequency was defined as background with > 50% of the record consisting of suppression/attenuation [9, 16]. When two or three frequency bands are equally prominent, the patients were categorized into the faster frequency group [16].

### Data collection

The investigators extracted clinical data from the prospectively collected web-based registry (KORHN-PRO). Data regarding age, sex, previous medical history, resuscitation profiles, TTM treatments, the presence of ocular reflexes including pupillary reflex and corneal reflex at ≥ 72 h, and motor component ≤ 3 of the Glasgow Coma Score at 48 and 72 h were collected. The primary end point was a poor neurologic outcome at 1 month after cardiac arrest. The CPC score was used to assess the neurological outcome. CPC 1 was defined as conscious and alert with good cerebral performance; CPC 2 as conscious and alert with moderate cerebral performance; CPC 3 as conscious with severe cerebral disability; CPC 4 as comatose or in a persistent vegetative state; and CPC 5 as brain dead or dead [17]. A CPC score of 3–5 being regarded as poor neurologic outcome. The actual causes of death were categorized into four groups: (1) cardiovascular cause, defined as a circulatory failure despite the use of vasoactive drugs, intra-aortic balloon pump, and extracorporeal membrane oxygenation or the development of new fatal arrhythmia; (2) cerebral cause, defined as a comatose state in the absence of sedation with evidence of severe hypoxic brain injury on brain computed tomography or brain magnetic resonance image or a diagnosis of brain death; (3) multiple organ failure, defined as a combination of cardiovascular or cerebral failure with respiratory or renal failure, and sepsis; and (4) others or unknown. The investigators also retrieved the awakening time of the study patients to further distinguish between the death after awakening and the neurological causes of death, the data about the withholding of the active treatment including no further examination or therapeutic interventions and no further cardiopulmonary resuscitation with maintaining then current treatment and the decision timing. The neurologic outcome of survivors was determined at 1 month by reviewing the electronic medical records of hospitalized patients or through standardized follow-up telephone interviews with the patient or a family member per the KORHN-PRO protocol. Each principal investigator of participating hospitals recorded the 1-month neurological outcome for the patients admitted to hospitals for more than one month. For the patients transferred to other hospitals or discharged to home, the independent data input commission investigated the survival rate and neurological outcome through standardized follow-up telephone interviews with the patient or a family member at 1 month and 6 months after cardiac arrest.

### Statistical analysis

Data of continuous variables were reported as median with interquartile range (IQR) because of their non-normal distribution using the Kolmogorov–Smirnov test. Data of categorical variables were presented as numbers and percentages. Comparisons of demographic and clinical characteristics, including resuscitation profiles, post-resuscitation treatments, and EEG findings, between the early and late EEG groups were performed using the Mann–Whitney U-test for continuous variables and the chi-square test or Fisher’s exact test for categorical variables, as appropriate. In the early and late EEG groups, we also compared the characteristics between patients with good and poor neurological outcomes. Sensitivity, specificity, positive predictive value (PPV) and negative predictive value (NPV), positive likelihood ratio, and negative likelihood ratio for poor neurological outcomes at 1 month were calculated using the binomial 95% confidence intervals (CIs). Two-tailed *p* values of < 0.05 were considered to indicate statistical significance. All statistical analyses were performed using IBM SPSS Statistics for Windows, version 21.0 (IBM Corp., Armonk, NY, USA), and MEDCALC software (Medcalc Software, version 9.2.1.0, Mariakerke, Belgium).

## Results

**Fig. 1:**
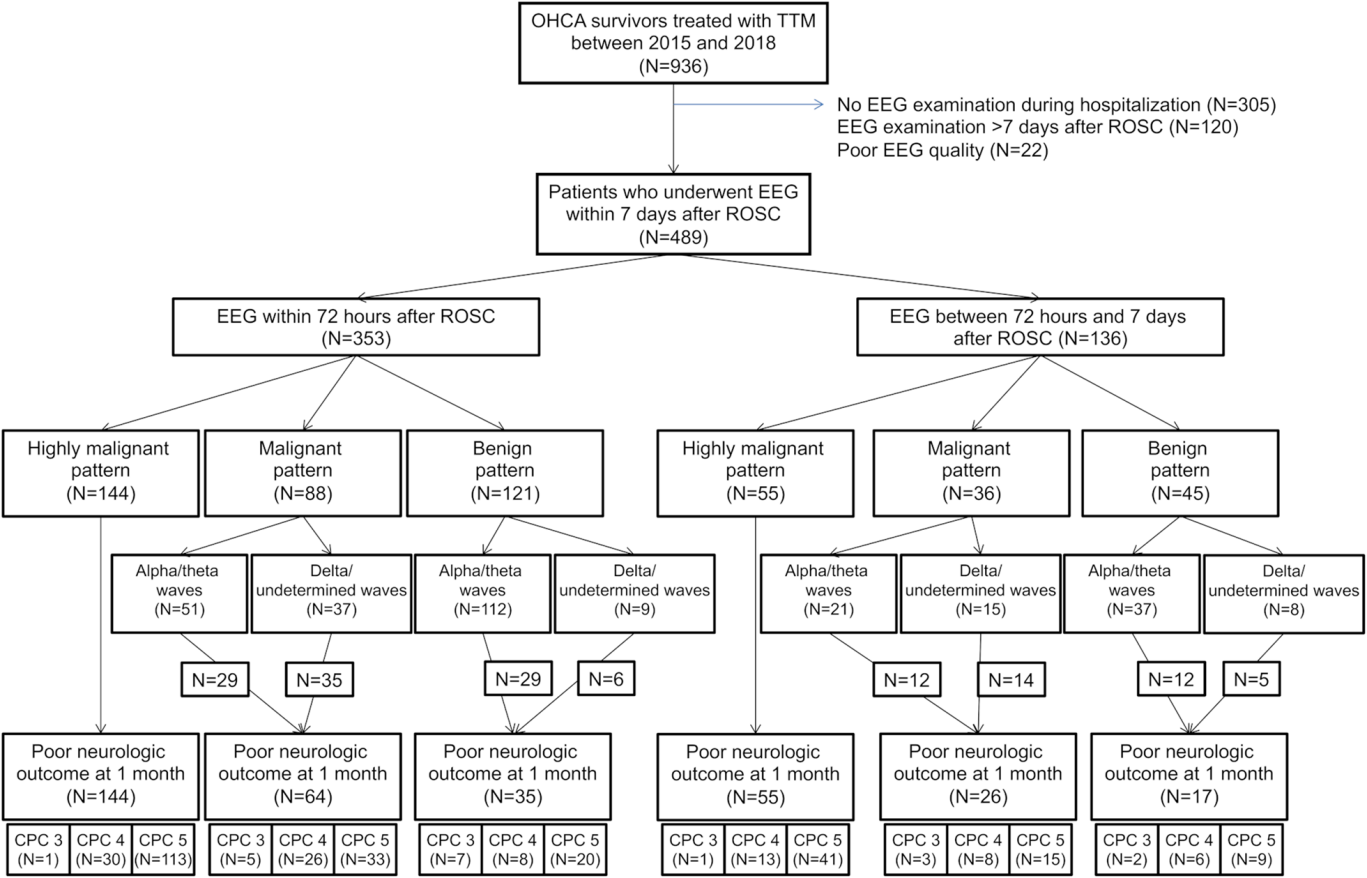
Flow diagram of the patient selection process. CPC, cerebral performance category; EEG, electroencephalography; OHCA, out-of-hospital cardiac arrest; ROSC, return of spontaneous circulation; TTM, targeted temperature management

A total of 936 non-traumatic comatose OHCA survivors with TTM were enrolled, and patients without standard EEG examination during hospitalization (*n* = 305), with EEG examination after 7 days since cardiac arrest (*n* = 120), and with poor EEG quality (*n* = 22) were excluded (Fig. 1). The remaining 489 patients who underwent standard EEG examination within 7 days since cardiac arrest were categorized into the early EEG group, i.e., EEG examination within 72 h after ROSC (*n* = 353, 72.2%), or the late EEG group, i.e., EEG examination between 72 h and 7 days (*n* = 115, 23.5%).

The clinical characteristics and neurologic outcome of patients according to the EEG timing are summarized in Table 1. The two groups were similar in terms of age, sex, previous medical history, resuscitation profiles, and neurologic outcome at 1 month and 6 months, except the target temperature of TTM. The median age of the cohort was 58.0 years, and 341 (69.7%) patients were male. The median time from ROSC to EEG examination was 31.5 h in the early EEG group and 90.8 h in the late EEG group. The rate of poor neurologic outcome at 1 month was 69.7% (341/489) and did not significantly differ between the early and late EEG groups (68.8% vs. 72.1%, *P* = 0.487). EEG findings, such as predominant frequency of background EEG and pattern classification, were not significantly different between the two groups (Table 2). Regardless of the EEG examination timing, the “highly malignant” pattern (40.7%) was the most prevalent one in the study patients, followed by the “benign” (33.9%) and “malignant” (25.4%) patterns.

**Table 1:** Demographic and clinical characteristics of the study patients according to electroencephalography timing

Characteristics	Total (*N* = 489)	Patients with EEG < 72 h (*N* = 353)	Patients with EEG of 72 h to 7 days (*N* = 136)	*P* value
Age, years	58.0 (46.0–69.0)	58.0 (46.0–69.0)	57.0 (46.3–67.0)	0.515
Male	341 (69.7%)	251 (71.1%)	90 (66.2%)	0.288
Previous medical history				
Hypertension	171 (35.0%)	127 (36.0%)	44 (32.4%)	0.451
Diabetes mellitus	99 (20.2%)	78 (22.1%)	21 (15.4%)	0.101
Acute myocardial infarction	25 (5.1%)	16 (4.5%)	9 (6.6%)	0.348
Congestive heart failure	18 (3.7%)	13 (3.7%)	5 (3.7%)	0.997
Chronic kidney disease	38 (7.8%)	30 (8.5%)	8 (5.9%)	0.333
Cardiac arrest characteristics				
Witnessed	313 (64.0%)	228 (64.6%)	85 (62.5%)	0.666
Bystander CPR	296 (60.5%)	212 (60.1%)	84 (61.8%)	0.729
Initial shockable rhythm	169 (34.6%)	127 (36.0%)	42 (30.9%)	0.288
No flow time, min	1.0 (0.0–6.0)	1.0 (0.0–6.0)	1.0 (0.0–7.0)	0.935
Resuscitation duration, min	24.0 (13.5–36.0)	24.0 (13.0–37.5)	24.0 (14.0–32.0)	0.48
Time from ROSC to TTM initiation, hours	3.2 (2.0–4.7)	3.2 (1.9–4.6)	3.3 (2.2–4.7)	0.599
Target temperature				0.049
33 °C	273 (55.8%)	185 (52.4%)	88 (64.7%)	
34–35 °C	160 (32.7%)	124 (35.1%)	36 (26.5%)	
36 °C	56 (11.5%)	44 (12.5%)	12 (8.8%)	
Time from ROSC to EEG examination, hours	46.6 (23.9–73.9)	31.5 (20.0–52.0)	90.8 (77.8–106.1)	< 0.001
Poor neurological outcome at 1 month	341 (69.7%)	243 (68.8%)	98 (72.1%)	0.487
Death at 1 month	231 (47.2%)	166 (47.0%)	65 (47.8%)	0.879
Poor neurological outcome at 6 months	335 (69.4%), *N* = 483	237 (68.3%), *N* = 347	98 (72.1%), *N* = 136	0.420
Death at 6 months	276 (57.1%), *N* = 483	197 (56.8%), *N* = 347	79 (58.1%), *N* = 136	0.793

Values are expressed as median (interquartile range) or *n* (%) as appropriate

CPR, cardiopulmonary resuscitation; ROSC, return of spontaneous circulation; TTM, targeted temperature management; EEG, electroencephalography

**Table 2:** Electroencephalography patterns and findings of the study patients according to electroencephalography timing

EEG findings	Total (*N* = 489)	Patients with EEG < 72 h (*N* = 353)	Patients with EEG of 72 h to 7 days (*N* = 136)	*P* value
EEG background frequency				0.220
Predominant alpha waves	106 (21.7%)	84 (23.8%)	22 (16.2%)	
Predominant theta waves	127 (26.0%)	87 (24.6%)	40 (29.4%)	
Predominant delta waves	90 (18.4%)	67 (19.0%)	23 (16.9%)	
Undetermined	166 (33.9%)	115 (32.6%)	51 (37.5%)	
Categorization				0.934
Highly malignant pattern	199 (40.7%)	144 (40.8%)	55 (40.4%)	
Malignant pattern	124 (25.4%)	88 (24.9%)	36 (26.5%)	
Benign	166 (33.9%)	121 (34.3%)	45 (33.1%)	
Highly malignant pattern				
Suppressed background without discharges	154 (31.5%)	106 (30.0%)	48 (35.3%)	0.261
Suppressed background with continuous periodic discharges	10 (2.0%)	8 (2.3%)	2 (1.5%)	0.733
Burst-suppression	35 (7.2%)	30 (8.5%)	5 (3.7%)	0.064
Malignant rhythmic or periodic features	67 (13.7%)	44 (12.5%)	23 (16.9%)	0.2
Periodic discharges (≥ 50%)	30 (6.1%)	19 (5.4%)	11 (8.1%)	0.264
Rhythmic spike-and-wave (≥ 50%)	28 (5.7%)	21 (5.9%)	7 (5.1%)	0.732
Unequivocal seizures or status epilepticus	53 (10.8%)	37 (10.5%)	16 (11.8%)	0.683
Malignant background	85 (17.4%)	64 (18.1%)	21 (15.4%)	0.527
Discontinuous (> 10% suppression)	16 (3.3%)	10 (2.8%)	6 (4.4%)	0.399
Low voltage (< 20 μV)	61 (12.5%)	47 (13.3%)	14 (10.3%)	0.365
Reversed anteroposterior gradient	14 (2.9%)	11 (3.1%)	3 (2.2%)	0.766

Values are expressed as number (%)

EEG, electroencephalography

### Predictive value of EEG findings for poor neurologic outcome stratified by EEG timing

Table 3 shows a comparison of the EEG findings and clinical examinations of the study patients in the early and late EEG groups according to the neurologic outcome at 1 month. Alpha and theta waves were more prevalent in the good neurologic outcome group of both the early and late EEG groups, and all patients with undetermined background frequncy had poor neurologic outcome in both the early (0% vs. 47.3%) and late (0% vs. 52.0%) EEG groups. Suppressed background without discharges was the most frequently reported finding among the “highly malignant” EEG pattern in both the early (106/144, 73.3%) and late (48/55, 87.3%) EEG groups. In the early EEG group, the malignant rhythmic or periodic features, defined as any presence of abundant periodic discharges; abundant rhythmic polyspike-/spike-/sharp-and-wave, and unequivocal electrographic seizure, were significantly more frequent for patients with the poor neurologic outcome (5.5% vs. 15.6%, *P* = 0.007). In contrast, malignant EEG background, defined as any presence of discontinuous background; low-voltage background; and reversed anterior–posterior gradient, did not significantly differ between patients with good and poor neurologic outcomes (16.4% vs. 18.9%, *P* = 0.562). In the late EEG group, there were no differences between patients with good and poor neurologic outcomes in terms of the prevalence of malignant rhythmic or periodic features (18.4% vs. 16.3%, *P* = 0.770) and malignant EEG background (7.9% vs. 18.4%, *P* = 0.129). Absent ocular reflexes at 72 h after ROSC and motor component of Glasgow Coma Score significantly differed between patients with good and poor neurologic outcomes.

**Table 3:** Electroencephalography findings and clinical examinations of the study patients in the early and late electroencephalography groups according to neurologic outcome at 1 month

EEG findings and clinical neurological examinations	Patients with EEG < 72 h (*N* = 353)			Patients with EEG of 72 h to 7 days (*N* = 136)		
	Good neurologic outcome (*n* = 110)	Poor neurologic outcome (*n* = 243)	*P* value	Good neurologic outcome (*n* = 38)	Poor neurologic outcome (*n* = 98)	*P* value
EEG background frequency			< 0.001			< 0.001
Dominant alpha waves	69 (62.7%)	15 (6.2%)		14 (36.8%)	8 (8.2%)	
Dominant theta waves	36 (32.7%)	51 (21.0%)		20 (52.6%)	20 (20.4%)	
Dominant delta waves	5 (4.5%)	62 (25.5%)		4 (10.5%)	19 (19.4%)	
Undetermined	0 (0%)	115 (47.3%)		0 (0%)	51 (52.0%)	
Categorization			< 0.001			< 0.001
Highly malignant pattern	0 (0%)	144 (59.3%)		0 (0%)	55 (56.1%)	
Malignant pattern	24 (21.8%)	64 (26.3%)		10 (26.3%)	26 (26.5%)	
Benign	86 (78.2%)	35 (14.4%)		28 (73.7%)	17 (17.3%)	
*Highly malignant pattern*	0 (0%)	144 (59.3%)	< 0.001	0 (0%)	55 (56.1%)	< 0.001
Suppressed background without discharges	0 (0%)	106 (43.6%)	< 0.001	0 (0%)	48 (49.0%)	< 0.001
Suppressed background with continuous periodic discharges	0 (0%)	8 (3.3%)	0.061	0 (0%)	2 (2.0%)	> 0.999
Burst-suppression	0 (0%)	30 (12.3%)	< 0.001	0 (0%)	5 (5.1%)	0.322
*Malignant rhythmic or periodic features*	6 (5.5%)	38 (15.6%)	0.007	7 (18.4%)	16 (16.3%)	0.770
Periodic discharges (≥ 50%)	2 (1.8%)	17 (7.0%)	0.046	3 (7.9%)	8 (8.2%)	> 0.999
Rhythmic spike-and-wave (≥ 50%)	3 (2.7%)	18 (7.4%)	0.085	1 (2.6%)	6 (6.1%)	0.673
Unequivocal seizures or status epilepticus	3 (2.7%)	34 (14.0%)	0.001	6 (15.8%)	10 (10.2%)	0.382
*Malignant background*	18 (16.4%)	46 (18.9%)	0.562	3 (7.9%)	18 (18.4%)	0.129
Discontinuous (> 10% suppression)	2 (1.8%)	8 (3.3%)	0.730	1 (2.6%)	5 (5.1%)	> 0.999
Low voltage (< 20 μV)	14 (12.7%)	33 (13.6%)	0.827	2 (5.3%)	12 (12.2%)	0.349
Reversed anteroposterior gradient	2 (1.8%)	9 (3.7%)	0.513	0 (0%)	3 (3.1%)	0.560
Absent pupillary reflex at ≥ 72 h after ROSC^a^	5 (4.7%), *n* = 107	158 (67.8%), *n* = 233	< 0.001	2 (5.3%), *n* = 38	62 (63.9%), *n* = 97	< 0.001
Absent corneal reflex at ≥ 72 h after ROSC^a^	16 (20.3%), *n* = 79	170 (85.9%), *n* = 198	< 0.001	5 (16.1%), *n* = 31	59 (79.7%), *n* = 74	< 0.001
Motor component of the Glasgow Coma Score^b^						
≤ 3 at 48 h after ROSC	83 (77.6%), *n* = 107	238 (98.8%), *n* = 241	< 0.001	28 (77.8%), *n* = 36	96 (99.0%), *n* = 97	< 0.001
≤ 3 at 72 h after ROSC	55 (50.5%), *n* = 109	233 (97.9%), *n* = 238	< 0.001	16 (45.7%), *n* = 35	96 (98.0%), *n* = 98	< 0.001

Values are expressed as number (%)

EEG, electroencephalography; ROSC, return of spontaneous circulation

^a^A total of 13 patients dead < 72 h after ROSC were categorized as absent ocular reflexes

^b^Two patients dead < 48 h after ROSC were categorized as motor component ≤ 3. Similarly, 13 patients dead < 72 h were categorized as motor component ≤ 3

### Impact of background frequency on the prognostic value of neurologic outcome

All patients with the “highly malignant” EEG pattern had a poor neurologic outcome. The “highly malignant” EEG patterns showed 100% specificity in both the early and late EEG groups but 59.3% and 56.1% sensitivity, respectively, in the early and late EEG groups (Table 4). The “highly malignant” or “malignant” EEG patterns showed 84.8% sensitivity and 77.0% specificity for the poor neurologic outcome. The combination of “highly malignant” or “malignant” EEG patterns with predominant delta and undetermined background EEG waves predicted the poor neurologic outcome with 70.4% sensitivity and 98.2% specificity in the early EEG group. Two patients with a false-positive result were categorized into “malignant” EEG pattern due to low-voltage background. In the late EEG group, one patient with malignant rhythmic or periodic feature and predominant delta waves had good neurologic outcome. The combination of EEG pattern and background frequency predicted poor neurologic outcome with 66.3% sensitivity and 97.4% specificity in the late EEG group. A total of 179 of 239 patients with a combination of EEG pattern and background frequency (74.9%) died within a month, and the major causes of death were cerebral cause (*n* = 92, 51.4%) and multiple organ failure (*n* = 47, 26.3%) (Table 5). Six patients (2.6%) were dead after awakening from the post-anoxic coma, and all of them did not have EEG of highly malignant or malignant patterns with delta or undetermined frequency. Withholding therapies including no therapeutic escalation or no cardiopulmonary resuscitation, i.e., do not resuscitate order, was occurred in 52 patients (10.6%), and the median time to withholding decision was 82.0 h.

**Table 4:** Predictive value of “highly malignant” and “malignant” electroencephalography patterns and dominant electroencephalography frequency for poor neurologic outcome at 1 month

Variables	EEG timing	True positive	False positive	True negative	False negative	Sensitivity (95% CI)	Specificity (95% CI)	PPV (95% CI)	NPV (95% CI)	Accuracy (95% CI)
Highly malignant pattern	< 72 h	144	0	110	99	59.3 (52.8–65.5)	100.0 (96.7–100.0)	100	52.6 (48.8–56.4)	72.0 (67.0–76.6)
	Between 72 h and 7 days	55	0	38	43	56.1 (45.7–66.1)	100.0 (90.8–100.0)	100	46.9 (41.4–52.5)	68.4 (59.9–76.1)
	< 7 days	199	0	148	142	58.4 (52.9–63.6)	100.0 (97.5–100.0)	100	51.0 (47.9–54.2)	71.0 (66.7–75.0)
Highly malignant or malignant patterns	< 72 h	208	24	86	35	85.6 (80.5–89.8)	78.2 (69.3–85.5)	89.7 (85.8–92.5)	71.1 (64.0–77.2)	83.3 (79.0–87.0)
	Between 72 h and 7 days	81	17	28	10	82.7 (73.7–89.6)	73.7 (56.9–86.6)	89.0 (82.5–93.3)	62.2 (50.7–72.5)	80.2 (72.5–86.5)
	< 7 days	289	34	114	52	84.8 (80.5–88.4)	77.0 (69.4–83.5)	89.5 (86.3–92.0)	68.7 (62.7–74.1)	82.4 (78.7–85.7)
Highly malignant or malignant patterns with delta or undetermined frequency	< 72 h	171	2	108	72	70.4 (64.2–76.0)	98.2 (93.6–99.8)	98.8 (95.6–99.7)	60.0 (55.2–64.6)	79.0 (74.4–83.2)
	Between 72 h and 7 days	65	1	37	33	66.3 (56.1–75.6)	97.4 (86.2–99.9)	98.5 (90.3–99.8)	52.9 (45.8–59.8)	75.7 (67.9–82.0)
	< 7 days	236	3	145	105	69.2 (64.0–74.1)	98.0 (94.2–99.6)	98.7 (96.2–99.6)	58.0 (54.0–61.9)	77.9 (74.0–81.5)

CI, confidence interval; EEG, electroencephalography; NPV, negative predictive value; PPV, positive predictive value

**Table 5:** The distribution of cerebral performance category score at 1 month and the cause of death

Variable	Total (*N* = 489)	Highly malignant or malignant patterns with delta or undetermined frequency	
		Yes (*N* = 239)	No (*N* = 250)
CPC 1	134 (27.4%)	2 (0.8%)	132 (52.8%)
CPC 2	14 (2.9%)	1 (0.4%)	13 (5.2%)
CPC 3	19 (3.9%)	4 (1.7%)	15 (6.0%)
CPC 4	91 (18.6%)	53 (22.2%)	38 (15.2%)
CPC 5	231 (47.2%)	179 (74.9%)	52 (20.8%)
Cause of death			
Cerebral cause	108 (46.8%)	92 (51.4%)	16 (30.8%)
Multiple organ failure	68 (29.4%)	47 (26.3%)	21 (40.4%)
Cardiovascular cause	25 (10.8%)	17 (9.5%)	8 (15.4%)
Others/ Unknown	30 (13.0%)	23 (12.8%)	7 (13.5%)
Death after awakening^a^	6 (2.6%)	0 (0%)	6 (11.5%)
Withholding therapies	52 (10.6%)	42 (17.6%)	10 (4.0%)
No therapeutic escalation	9 (1.8%)	8 (3.3%)	1 (0.4%)
No cardiopulmonary resuscitation	41 (8.4%)	32 (13.4%)	9 (3.6%)
Time to withholding of active treatment, hours	82.0 (40.3–118.3)	89.5 (39.5–125.0)	78.0 (35.8–97.0)

Values are expressed as number (%) and median (interquartile range)

CPC, cerebral performance category

^a^A total of 6 patients were dead after awakening due to cardiovascular cause (*n* = 2), multiple organ failure (*n* = 1) and others/unknown cause (*n* = 3)

## Discussion

In this multicenter study, the “highly malignant” EEG pattern was the most prevalent one (40.7%; 199/489), and all patients with this EEG pattern had poor neurologic outcome, regardless of EEG measurement time. However, patients with the “highly malignant” or “malignant” EEG pattern showed 77.0% specificity and 89.5% PPV for poor neurologic outcome. We found that EEG background frequency of delta and undetermined waves had a prognostic value and that the combination of the “highly malignant” or “malignant” EEG pattern with background frequency of delta and undetermined waves increased the specificity and PPV to up to 98.0% and 98.7%, respectively. Therefore, we documented the importance of background frequency in the assessment of neuro-prognostication in comatose OHCA survivors with TTM.

Previously, the unfavorable patterns were grouped as “highly malignant” or “malignant.” The “highly malignant” patterns included suppressed background with or without superimposed periodic discharges and burst suppression [8, 10]. The specificity of the “highly malignant” EEG pattern for poor outcomes was 90.6–100% [8, 11, 12, 18, 19]. Bongiovanni et al. conducted their investigation using standardized EEG classification for neurologic outcome and showed the presence of a “highly malignant” EEG pattern on day 2, which was very specific (specificity: 99.5%; 95% CI, 97.4%–99.9%) for poor prognosis in patients with an initial indeterminate outcome [20]. Recently, a sub-study of a TTM trial, including 103 patients with intermittent EEG, demonstrated that the “highly malignant” EEG pattern reliably predicted poor outcomes with a high specificity, and this was confirmed in a small study with 62 patients that the presence of the “highly malignant” EEG pattern was predictive of a poor neurological outcome with 100% specificity and 42% sensitivity [11]. A recent systemic review on the prediction of poor neurological outcome in comatose cardiac arrest survivors also revealed that the false positive rate for “highly malignant” EEG patterns achieved 0% in most studies regardless of EEG examination timing during the first 72 h after ROSC [15]. However, in contrast, the sensitivity progressively decreased during the first 72 h after ROSC [15]. In line with the findings of recent reports, we found that all patients with the “highly malignant” EEG pattern had a poor neurologic outcome in both the early and late EEG groups, with 100% specificity but 59.3% and 56.1% sensitivity in the early and late EEG groups, respectively.

Previous literature about the neurological prognostication using EEG of the poor neurologic outcome has been susceptible to errors caused by the following factors: lack of standardized terminology and definitions, relatively small sample sizes with single-center study design, the risk of self-fulfilling prophecy, and lack of the adjustment for effects of medications [21, 22]. This study is the multicenter validation study with a relatively large sample size on the prognostic performance for the proposed three standardized EEG patterns in comatose OHCA survivors with TTM. WLST had been prohibited until February 2018 and did not occur for the study patients, which could decrease the bias [21]. Our study results added evidence for the usefulness of EEG pattern classification for prognostication in comatose cardiac arrest survivors, consistent with the recently released guidelines from the European Resuscitation Council [2]. However, the accuracy for patients with the “malignant” EEG pattern has been inconsistent, with a higher false-positive rate than that in the “highly malignant” EEG pattern [11, 12, 15]. This finding has been reconfirmed with the results of the present study that patients with the “highly malignant” or “malignant” EEG pattern showed 84.8% sensitivity, 77.0% specificity, and 89.5% PPV for poor neurologic outcome. It is clear that further research is needed to improve the prognostic value of patients with the “malignant” EEG pattern.

The main aspects of EEG assessment are the background activity, superimposed discharges, and reactivity. However, EEG background frequencies, measured in Hertz and classified as beta, alpha, theta, delta, and undetermined, are one of the neglected aspects in post-cardiac arrest patients. Our previous study showed that the prevalence of predominant delta and undetermined background frequency was 8.8% (15/170) and 45.3% (77/170), respectively [16]. A total of eight of 92 patients (8.7%) with predominant delta and undetermined background frequency achieved the good neurologic outcome at 6-month, whereas 60 patients (76.9%) with predominant alpha and theta background frequency did [16]. The background EEG frequency with predominant alpha and theta waves was a powerful predictor (adjusted odds ratio for a good neurologic outcome of 13.030) with high sensitivity (86.21%) and NPV (91.30%) [16]. In the present study, 90 patients (18.4%) showed predominant delta background frequency, and 166 patients (33.9%) had undetermined background frequency. Consistent with the previous study, 3.5% (9/256) of patients with background EEG frequency with predominant delta waves or undetermined waves had a good neurologic outcome. The combination of the “highly malignant” or “malignant” EEG pattern with background frequency of delta waves or undetermined increased the specificity and PPV to up to 98.0% and 98.7%, respectively. Also, death after awakening did not occur in patients with the combination of the “highly malignant” or “malignant” EEG pattern with background frequency of delta waves or undetermined, which indicated that the indirect outcome and misclassification of a neurological outcome less likely occurred [21].

In addition to an insufficient sample size for EEG pattern analysis, as shown in previous studies, the EEG assessment time point has been also an important factor that influences the efficiency of prediction. Because EEG background pattern categories were not significantly associated with outcome within the first 12 h of cardiac arrest, the European Resuscitation Council currently suggests the use of these EEG patterns examined at least after 24 h after cardiac arrest [2]. In this sub-study of the “TTM for 48 vs. 24 trial,” there were no significant differences between prognostication at 24 h and that at 48 h measured in terms of the specificity and sensitivity of EEG categories [18]. Additionally, our study showed that the prognostic values of EEG background pattern categories did not differ significantly between the post-resuscitation phase, i.e., < 72 h after ROSC, and the early hospitalization phase, i.e., 72 hours7 days after ROSC. The “highly malignant” pattern predicted the poor neurologic outcome with a high specificity regardless of EEG measurement time.

This study had several limitations. First, although all patients were treated in accordance with the latest guidelines, the potential differences among the institutions were not controlled. Second, despite using the recommended protocol for EEG examination, this study excluded a large proportion of patients owing to missing EEG data within 7 days after ROSC, and the risk of selection bias is inevitable. We compared the clinical characteristics and neurologic outcomes between the study patients and those excluded from our study (see Additional file 3). The included patients were younger (median, 58.0 vs. 61.0 years; *P* = 0.002), but had less frequent witnessed cardiac arrest (64.0% vs. 71.6%; *P* = 0.013) and longer time from ROSC to TTM initiation (median, 3.2 vs. 2.8 h, *P* = 0.002). The rate of the poor neurologic outcome at 1 month was 71.0% (665/936) and did not significantly differ between the two groups (72.5% vs. 69.7%, *P* = 0.354). Third, we did not assess the unreactive EEG finding, one of the malignant EEG patterns, owing to the lack of standardization of reactivity testing. This would have affected the predictive power of the malignant EEG patterns. However, recent guidelines also described the limitation of EEG background reactivity that there is no generally acknowledged consensus for the reactivity testing itself and its interpretation [2, 15, 23]. Fourth, the single intermittent EEG examination would be insufficient to identify all the malignant EEG features which might have been intermittent or have not emerged at the time of EEG recording. In this study, one board-certified epileptologist investigated the qualitative EEG evaluation. Although the investigator performed the EEG interpretation in a blinded to all clinical information, a single EEG rater limited the reliability of our results and could bring the detection bias. Finally, the variability in EEG timing and variation in the protocol, including ongoing sedation or anti-epileptic medication, could be another significant limitation of our study. However, our heterogeneous EEG timing during TTM reflected the real-world situation, and the results can therefore be generalized to other settings.

## Conclusions

In conclusion, the “highly malignant” EEG pattern predicted poor neurologic outcome with a high specificity regardless of EEG measurement time. We found that the predictive value of the EEG patterns could be improved when combined with the background frequency of delta waves/undetermined, which suggests that EEG background frequency has practical implications to assist in neuro-prognostication with the proposed EEG pattern classification. Further studies about the predictive value of “highly malignant EEG or malignant EEG pattern with background frequency of delta waves or undetermined” in a multimodal approach for comatose cardiac arrest patients will be needed.

## Supplementary Information

**Additional file 1:** Representative EEG of highly malignant (A-C) and malignant (D-E) pattern. (A) Highly malignant suppressed background (amplitude < 10 μV, 100% of the recording) without discharges; (B) Highly malignant suppressed background with superimposed continuous periodic discharges; (C) Highly malignant burst-suppression (periods of suppression with amplitude < 10 μV constituting > 50% of the recording) without discharges; (D) Malignant periodic or rhythmic patterns (abundant periodic discharges); (E) Malignant background (low-voltage with amplitude < 20 μV)

**Additional file 2:** Example of predominant background electroencephalography frequency. (A) predominant alpha waves; (B) predominant theta waves; (C) predominant delta waves; (D) undetermined background electroencephalography.

**Additional file 3:** Comparison of demographic and clinical characteristics between the patients with and without electroencephalography within 7 days.

## Data Availability

The datasets used and/or analyzed during the current study are available from the corresponding author on reasonable request.

## Abbreviations

ACNS: American Clinical Neurophysiology Society
CI: Confidence interval
CPC: Cerebral performance category
EEG: Electroencephalography
IQR: Interquartile range
KORHN-PRO: Korean Hypothermia Network prospective registry
NPV: Negative predictive value
OHCA: Out-of-hospital cardiac arrest
PPV: Positive predictive value
ROSC: Return of spontaneous circulation
TTM: Targeted Temperature Management
WLST: Withdrawal of life-sustaining therapy

## References

[1] Merchant RM, Topjian AA, Panchal AR, Cheng A, Aziz K, Berg KM, Lavonas EJ, Magid DJ (2020). Part 1: Executive Summary: 2020 American Heart Association Guidelines for Cardiopulmonary Resuscitation and Emergency Cardiovascular Care. Circulation.

[2] Nolan JP, Sandroni C, Böttiger BW, Cariou A, Cronberg T, Friberg H, Genbrugge C, Haywood K, Lilja G, Moulaert VRM (2021). European Resuscitation Council and European Society of Intensive Care Medicine guidelines 2021: post-resuscitation care. Intensive Care Med.

[3] Friberg H, Cronberg T, Dünser MW, Duranteau J, Horn J, Oddo M (2015). Survey on current practices for neurological prognostication after cardiac arrest. Resuscitation.

[4] Jordan KG (2004). Emergency EEG and continuous EEG monitoring in acute ischemic stroke. J Clin Neurophysiol.

[5] Muhlhofer W, Szaflarski JP (2018). Prognostic value of EEG in patients after cardiac arrest—an updated review. Curr Neurol Neurosci Rep.

[6] Rittenberger JC, Weissman A, Baldwin M, Flickinger K, Repine MJ, Guyette FX, Doshi AA, Dezfulian C, Callaway CW, Elmer J (2019). Preliminary experience with point-of-care EEG in post-cardiac arrest patients. Resuscitation.

[7] Westhall E, Rosén I, Rossetti AO, van Rootselaar AF, Wesenberg Kjaer T, Friberg H, Horn J, Nielsen N, Ullén S, Cronberg T (2015). Interrater variability of EEG interpretation in comatose cardiac arrest patients. Clin Neurophysiol.

[8] Westhall E, Rossetti AO, van Rootselaar AF, Wesenberg Kjaer T, Horn J, Ullén S, Friberg H, Nielsen N, Rosén I, Åneman A (2016). Standardized EEG interpretation accurately predicts prognosis after cardiac arrest. Neurology.

[9] Hirsch LJ, Fong MWK, Leitinger M, LaRoche SM, Beniczky S, Abend NS, Lee JW, Wusthoff CJ, Hahn CD, Westover MB (2021). American clinical neurophysiology society's standardized critical care EEG terminology: 2021 version. J Clin Neurophysiol.

[10] Westhall E, Rosén I, Rossetti AO, van Rootselaar AF, Kjaer TW, Horn J, Ullén S, Friberg H, Nielsen N, Cronberg T (2014). Electroencephalography (EEG) for neurological prognostication after cardiac arrest and targeted temperature management; rationale and study design. BMC Neurol.

[11] Backman S, Cronberg T, Friberg H, Ullén S, Horn J, Kjaergaard J, Hassager C, Wanscher M, Nielsen N, Westhall E (2018). Highly malignant routine EEG predicts poor prognosis after cardiac arrest in the Target Temperature Management trial. Resuscitation.

[12] Lilja L, Joelsson S, Nilsson J, Lindgren S, Rylander C (2021). Application of a standardized EEG pattern classification in the assessment of neurological prognosis after cardiac arrest: a retrospective analysis. Resuscitation.

[13] Becker LB, Aufderheide TP, Geocadin RG, Callaway CW, Lazar RM, Donnino MW, Nadkarni VM, Abella BS, Adrie C, Berg RA (2011). Primary outcomes for resuscitation science studies: a consensus statement from the American Heart Association. Circulation.

[14] Callaway CW, Donnino MW, Fink EL, Geocadin RG, Golan E, Kern KB, Leary M, Meurer WJ, Peberdy MA, Thompson TM (2015). Part 8: post-cardiac arrest care: 2015 American Heart Association guidelines update for cardiopulmonary resuscitation and emergency cardiovascular care. Circulation.

[15] Sandroni C, D'Arrigo S, Cacciola S, Hoedemaekers CWE, Kamps MJA, Oddo M, Taccone FS, Di Rocco A, Meijer FJA, Westhall E (2020). Prediction of poor neurological outcome in comatose survivors of cardiac arrest: a systematic review. Intensive Care Med.

[16] Kim YJ, Kim MJ, Koo YS, Kim WY (2020). Background frequency patterns in standard electroencephalography as an early prognostic tool in out-of-hospital cardiac arrest survivors treated with targeted temperature management. J Clin Med.

[17] Jennett B, Bond M (1975). Assessment of outcome after severe brain damage. Lancet.

[18] Duez CHV, Johnsen B, Ebbesen MQ, Kvaløy MB, Grejs AM, Jeppesen AN, Søreide E, Nielsen JF, Kirkegaard H (2019). Post resuscitation prognostication by EEG in 24 vs 48 h of targeted temperature management. Resuscitation.

[19] Rossetti AO, Tovar Quiroga DF, Juan E, Novy J, White RD, Ben-Hamouda N, Britton JW, Oddo M, Rabinstein AA (2017). Electroencephalography predicts poor and good outcomes after cardiac arrest: a two-center study. Crit Care Med.

[20] Bongiovanni F, Romagnosi F, Barbella G, Di Rocco A, Rossetti AO, Taccone FS, Sandroni C, Oddo M (2020). Standardized EEG analysis to reduce the uncertainty of outcome prognostication after cardiac arrest. Intensive Care Med.

[21] Geocadin RG, Callaway CW, Fink EL, Golan E, Greer DM, Ko NU, Lang E, Licht DJ, Marino BS, McNair ND (2019). Standards for studies of neurological prognostication in comatose survivors of cardiac arrest: a scientific statement from the American Heart Association. Circulation.

[22] Panchal AR, Bartos JA, Cabañas JG, Donnino MW, Drennan IR, Hirsch KG, Kudenchuk PJ, Kurz MC, Lavonas EJ, Morley PT (2020). Part 3: adult basic and advanced life support: 2020 American Heart Association guidelines for cardiopulmonary resuscitation and emergency cardiovascular care. Circulation.

[23] Admiraal MM, van Rootselaar AF, Horn J (2017). Electroencephalographic reactivity testing in unconscious patients: a systematic review of methods and definitions. Eur J Neurol.

