# Supplementary figures and images for: Background frequency can enhance the prognostication power of EEG patterns categories in comatose cardiac arrest survivors: a prospective, multicenter, observational cohort study
Source: Crit Care. 2021 Nov 17;25:398. doi: 10.1186/s13054-021-03823-y (PMC8596386; doi:10.1186/s13054-021-03823-y)

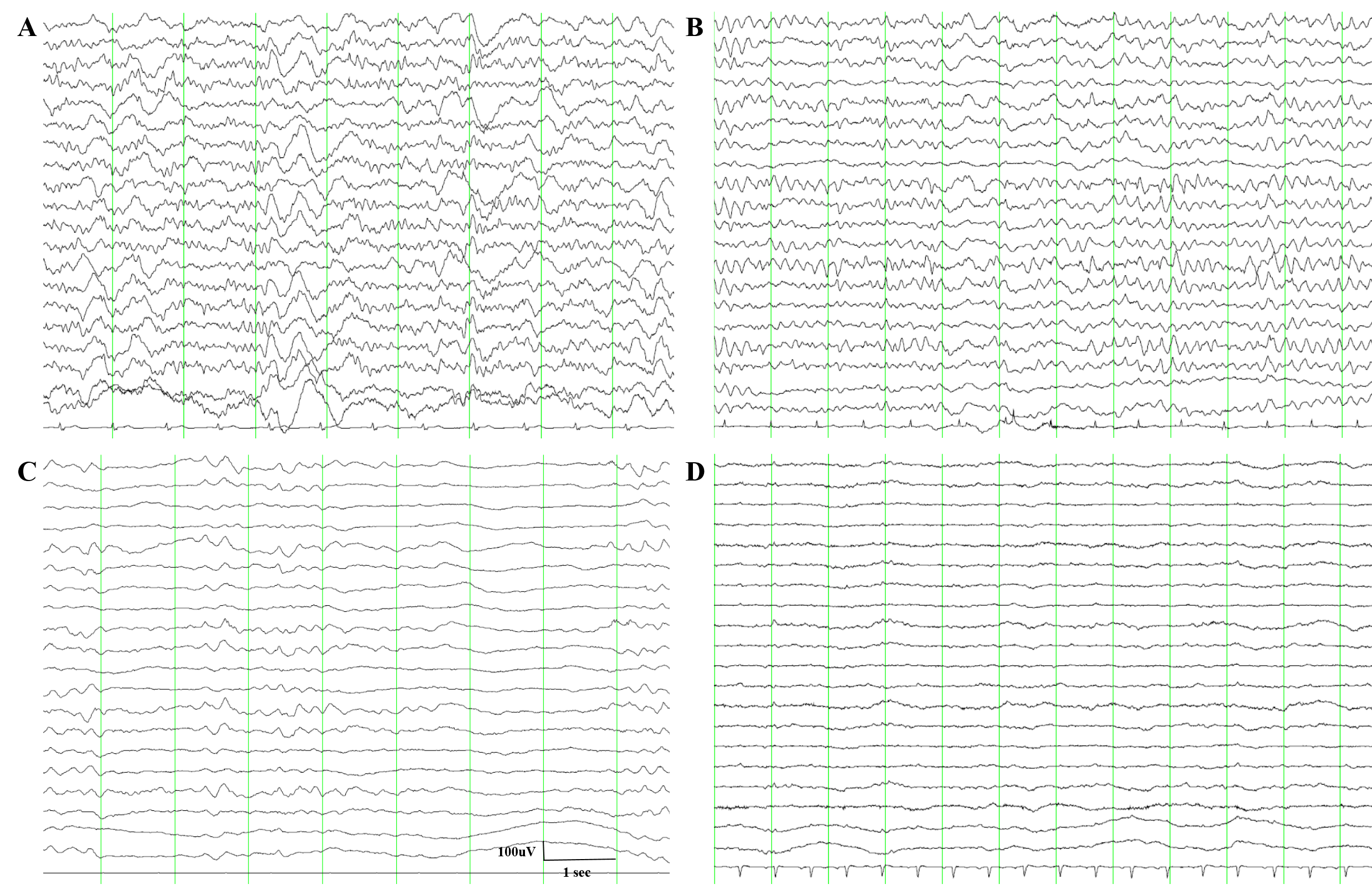

Supplement: Supplementary file 2 — Additional file 2: Example of predominant background electroencephalography frequency. (A) predominant alpha waves; (B) predominant theta waves; (C) predominant delta waves; (D) undetermined background electroencephalography. [file 13054_2021_3823_MOESM2_ESM.tif]
