# Supplementary material for: Background frequency can enhance the prognostication power of EEG patterns categories in comatose cardiac arrest survivors: a prospective, multicenter, observational cohort study
Source: Crit Care. 2021 Nov 17;25:398. doi: 10.1186/s13054-021-03823-y (PMC8596386; doi:10.1186/s13054-021-03823-y)
Supplement: Supplementary file 3 — Additional file 3: Comparison of demographic and clinical characteristics between the patients with and without electroencephalography within 7 days. [file 13054_2021_3823_MOESM3_ESM.docx]

Additional File 3. Comparison of demographic and clinical characteristics between the patients with and without electroencephalography within 7 days

| Characteristics | Total patients of  14 hospitals in  registry (N=936) | Patients with EEG within 7 days  (N=489) | Patients without  EEG within 7 days  (N=447) | P  value |
| --- | --- | --- | --- | --- |
| Age, years | 59.0 (47.3–72.0) | 58.0 (46.0–69.0) | 61.0 (49.0–73.0) | 0.002 |
| Male | 659 (70.4%) | 341 (69.7%) | 318 (71.1%) | 0.638 |
| Previous medical history |  |  |  |  |
| Hypertension | 326 (34.8%) | 171 (35.0%) | 155 (34.7%) | 0.925 |
| Diabetes mellitus | 210 (22.4%) | 99 (20.2%) | 111 (24.8%) | 0.093 |
| Acute myocardial infarction | 50 (5.3%) | 25 (5.1%) | 25 (5.6%) | 0.744 |
| Congestive heart failure | 31 (3.3%) | 18 (3.7%) | 13 (2.9%) | 0.509 |
| Chronic kidney disease | 68 (7.3%) | 38 (7.8%) | 30 (6.7%) | 0.533 |
| Cardiac arrest characteristics |  |  |  |  |
| Witnessed | 633 (67.6%) | 313 (64.0%) | 320 (71.6%) | 0.013 |
| Bystander CPR | 582 (62.2%) | 296 (60.5%) | 286 (64.0%) | 0.277 |
| Initial shockable rhythm | 308 (32.9%) | 165 (33.7%) | 143 (32.0%) | 0.569 |
| No flow time, min | 1.0 (0.0–7.0) | 1.0 (0.0–6.0) | 1.0 (0.0–7.0) | 0.959 |
| Resuscitation duration, min | 26.0 (16.0–38.0) | 24.0 (13.5–36.0) | 28.0 (17.0–40.0) | 0.024 |
| Time from ROSC to TTM initiation, hours | 3.0 (1.8–4.7) | 3.2 (2.0–4.7) | 2.8 (1.7–4.2) | 0.002 |
| Target temperature |  |  |  | 0.001 |
| 33 °C | 601 (64.2%) | 273 (55.8%) | 328 (73.4%) |  |
| 34-35 °C | 219 (23.4%) | 160 (32.7%) | 59 (13.2%) |  |
| 36 °C | 116 (12.4%) | 56 (11.5%) | 60 (13.4%) |  |
| Poor neurological outcome at 1 month | 665 (71.0%) | 341 (69.7%) | 324 (72.5%) | 0.354 |

Values are expressed as median (interquartile range) or n (%) as appropriate.

Abbreviations: CPR, cardiopulmonary resuscitation; ROSC, return of spontaneous circulation; TTM, targeted temperature management; EEG, electroencephalography.
